# Myricetin Attenuates Depressant-Like Behavior in Mice Subjected to Repeated Restraint Stress

**DOI:** 10.3390/ijms161226102

**Published:** 2015-11-30

**Authors:** Zegang Ma, Guilin Wang, Lin Cui, Qimin Wang

**Affiliations:** Department of Physiology, Medical College of Qingdao University, Shandong Provincial Collaborative Innovation Center for Neurodegenerative Disorders, Qingdao 266071, China; 18606321711@163.com (G.W.); 13685325208@126.com (L.C.); wangqimin12345@126.com (Q.W.)

**Keywords:** chronic stress, myricetin, depression, anti-oxidation, brain-derived neurotrophic factor

## Abstract

Increasing evidence has shown that oxidative stress may be implicated in chronic stress-induced depression. Several flavonoids with anti-oxidative effects have been proved to be anti-depressive. Myricetin is a well-defined flavonoid with the anti-oxidative, anti-inflammatory, anti-apoptotic, and neuroprotective properties. The aim of the present study is to investigate the possible effects of chronic administration of myricetin on depressant-like behaviors in mice subjected to repeated restraint (4 h/day) for 21 days. Our results showed that myricetin administration specifically reduced the immobility time in mice exposed to chronic stress, as tested in both forced swimming test and tail suspension test. Myricetin treatment improved activities of glutathione peroxidase (GSH-PX) in the hippocampus of stressed mice. In addition, myricetin treatment decreased plasma corticosterone levels of those mice subjected to repeated restraint stress. The effects of myricetin on the brain-derived neurotrophic factor (BDNF) levels in hippocampus were also investigated. The results revealed that myricetin normalized the decreased BDNF levels in mice subjected to repeated restraint stress. These findings provided more evidence that chronic administration of myricetin improves helpless behaviors. The protective effects of myricetin might be partially mediated by an influence on BDNF levels and might be attributed to myricetin-mediated anti-oxidative stress in the hippocampus.

## 1. Introduction

Depression is a common and life-threatening illness. It has a significant incidence in the population [1]. Numerous antidepressant drugs are now available presumably acting on different targets. Although approximately two-thirds of the depressed patients benefit from the current treatments, long-term effects are still disappointing [2]. The search for novel therapeutic compounds that can alleviate depression have been extensively explored over the past decades, in which herbal medicines are considered to be promising alternates [3]. Meanwhile, therapeutic potentials of a large number of herbal medicines have been investigated in a great variety of animal models [4].

Recently, growing evidence indicates that some natural anti-oxidative compounds serve as antidepressants due to their anti-oxidative and neuroprotective activities. Chronic administration of baicalein, a plant-derived active flavonoid extracted in the root of *Scutellaria baicalensis*, decreased depressant-like behavior in rats received repeated restraint stress [5]. Resveratrol, a non-flavonoid polyphenol antioxidant extracted from red grapes, also showed antidepressant effects in an animal model of depression by restoring the expressions of brain-derived neurotrophic factor (BDNF) in both hippocampus and frontal cortex [6]. The selected crude extracts from *Pilea microphylla* also showed an antidepressant effect. Further studies have demonstrated that the main contents of the crude extracts are flavonols.

Myricetin, which belongs to the large natural antioxidants group of flavonols, are widely presented in our daily food, teas, fruits, and medical herbs [7]. Previous studies have demonstrated that myricetin has an antioxidant activities and may prevent the decreases in the activities of antioxidant enzymes [8]. Studies have shown that myricetin inhibited age-related cognition declines [9] and protected against 6-hydroxydopamine (6-OHDA) and 1-methyl-4-phenyl-1,2,3,6-tetrahydropyridine (MPTP) induced neurotoxicity both *in vivo* and *in vitro* [10,11]. Therefore, the natural flavonol myricetin represents a great variety of promising agents with neuroprotective properties. Although the anti-oxidative, anti-inflammatory, and neuroprotective effects of myricetin have been well established, no clear evidence has shown that myricetin is anti-depressive. Therefore, in this study, we investigated the behavioral effects of myricetin administration on mice subjected to repeated restraint stress and explored the possible mechanisms involved.

## 2. Results

### 2.1. Effects of Myricetin on Repeated Restraint Stress Induced Depressant-Like Behaviors in Mice

**Figure 1:**
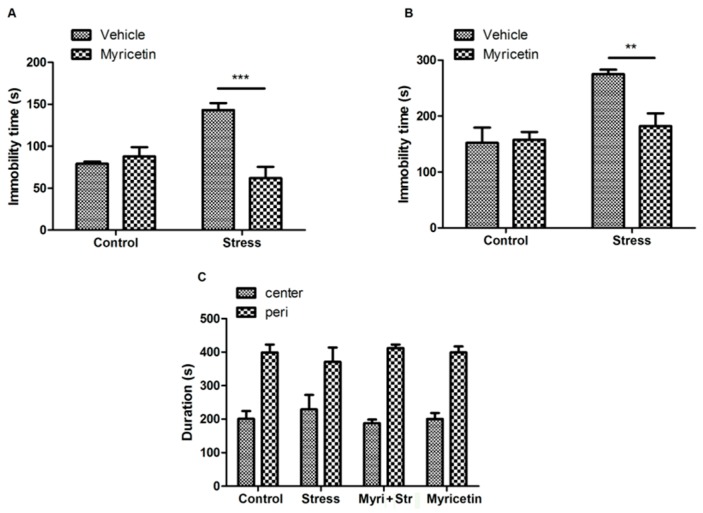
Effects of myricetin on repeated restraint stress-induced depressant-like behavior in mice. (**A**) Immobility time during the forced swimming test showing that myricetin treatment reversed the increase in the immobility time of the mice subjected to repeated restraint stress. Regular two-way Analysis of Variance (ANOVA), drug(myricetin) × treatment(stress) interaction *F*_(1,23)_ = 19.44, *p* < 0.001; significant drug variation *F*_(1,23)_ = 12.68, *p* < 0.01, Boferroni post-test, *** *p* < 0.001 significant difference between groups; (**B**) immobility time during the tail suspension test showing that myricetin treatment reversed the increase in the immobility time of the mice subjected to repeated restraint stress. Regular two-way ANOVA, drug(myricetin) × treatment(stress) interaction *F*_(1,23)_ = 6.85, *p* < 0.05; significant treatment variation *F*_(1,23)_ = 15.28, *p* < 0.001; significant drug variation *F*_(1,23)_ = 5.41, *p* < 0.05, Boferroni post-test, ** *p* < 0.001 significant difference between groups; and (**C**) the open field analysis showing that mice received different treatment spent similar time in the central (50%) area and the peripheral (50%) area of tested arena.

After exposure to repeated restraint stress for 21 days, the mice showed a significant increase in the immobility time compared to controls, as tested in the forced swimming test (FST) (Figure 1A). Administration of myricetin in 50 mg/kg for 21 days completely reversed the increase in the immobility time of the stressed mice, indicating that myricetin plays a certain anti-depressant effect on restrained mice. Importantly, we did not observe significant effects of myricetin on non-restraint control mice (Figure 1A). We further tested the anti-depressant effect of myricetin by using tail suspension test (TST), a well-accepted and antidepressant-sensitive behavioral task similar to FST. As shown in Figure 1B, myricetin treatment also remarkably decreased the immobility time of the stressed mice in TST.

To confirm that the antidepressant-like effect of myricetin is not due to any psychostimulant, the mice received an open field test 24 h after TST. The mice subjected to repeated restraint stress spent similar time in the center of the field compared to that of the controls, suggesting that repeated restraint stress does not change anxiety of the mice. Consistently, open field analysis indicated that myricetin treatment does not affect locomotor activity or anxiety of the mice (Figure 1C).

### 2.2. Effects of Myricetin on Plasma Corticosterone Levels in Mice Subjected to Repeated Restraint Stress

**Figure 2:**
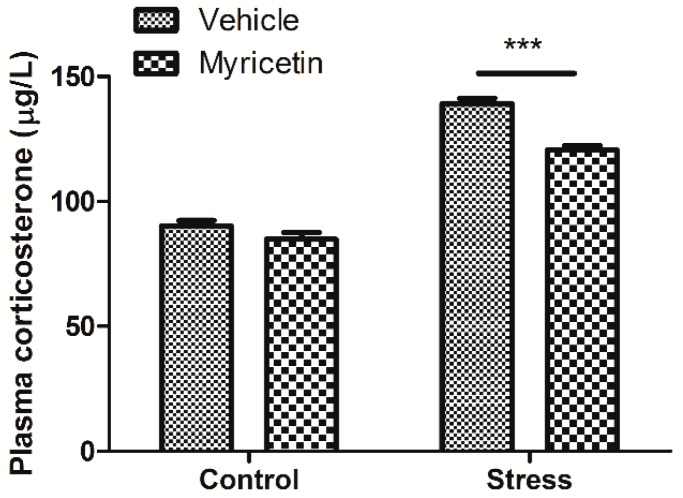
Effects of myricetin on the plasma corticosterone levels of mice subjected to repeated restraint stress for 21 consecutive days. Myricetin treatment reversed the increase in the plasma corticosterone of the mice subjected to repeated restraint stress. Regular two-way ANOVA, drug(myricetin) × treatment(stress) interaction *F*_(1,24)_ = 8.68, *p* < 0.01; significant treatment variation *F*_(1,24)_ = 353.07, *p* < 0.001; significant drug variation *F*_(1,24)_ = 28.17, *p* < 0.001, Boferroni post-test, *** *p* < 0.001 significant difference between groups.

It has been shown that chronic exposure to restraint stress may disrupt the hypothalamic-pituitary-adrenal (HPA) axis and induce the elevation of plasma corticosterone. We next measured the corticosterone levels in plasma of the mice. We found that the corticosterone contents in plasma of the mice exposed to repeated restraint stress were significantly higher than that of the controls, indicating that repeated restraint stimuli causes stress and depression. More importantly, our results showed that the corticosterone levels in the plasma of repeated restraint mice were significantly decreased by myricetin administration when compared to the control mice receiving vehicle treatment (Figure 2). Again, myricetin alone had no detectable effect on the plasma corticosterone levels of non-restraint control mice.

### 2.3. Effects of Myricetin on GSH-PX and SOD Activities in Mice Subjected to Repeated Restraint Stress

**Figure 3:**
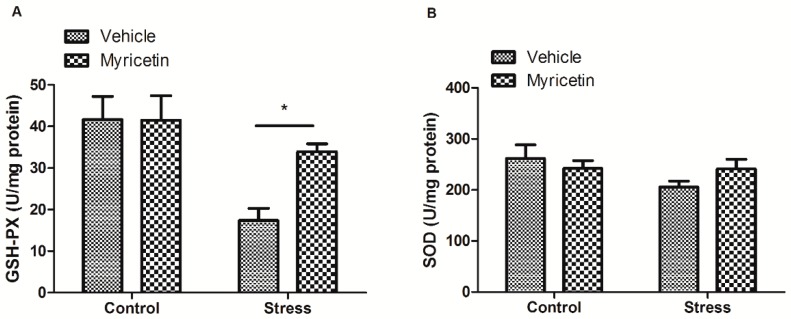
Effects of myricetin on the activities of glutathione peroxidase (GSH-PX) and superoxide dismutase (SOD) in the hippocampus of mice subjected to repeated restraint stress for 21 consecutive days. (**A**) Myricetin treatment reversed the decrease in the GSH-PX activity of the mice subjected to repeated restraint. Regular two-way ANOVA, significant treatment variation *F*_(1,23)_ = 12.21, *p* < 0.01, Boferroni post-test, * *p* < 0.05 significant difference between groups; and (**B**) the SOD activity in hippocampus of mice subjected to stress exhibited a tendency of decrease, as compared to the control group, however, was not significant.

Previous studies indicated that repeated restraint stress may induce robust increase in basal oxidative stress. Next, we measured the oxidative state in hippocampus of the mice. We found that mice exposed to repeated restraint stress exhibit remarkable reduction in the hippocampal GSP-PX activity, as compared to the control group (Figure 3A). Such decrease in hippocampal GSP-PX activity induced by restraint stress was partially restored by myricetin treatment (Figure 3A). Consistently, we did not observe any effects of myricetin on hippocampal GSP-PX activity in non-restraint control mice. The SOD activity in hippocampus in mice treated with repeated restraint exhibited a tendency of decrease, as compared to the controls, however, was not significant (Figure 3B).

### 2.4. Effects of Myricetin on the Brain-Derived Neurotrophic Factor (BDNF) Levels in Mice Subjected to Repeated Restraint Stress

**Figure 4:**
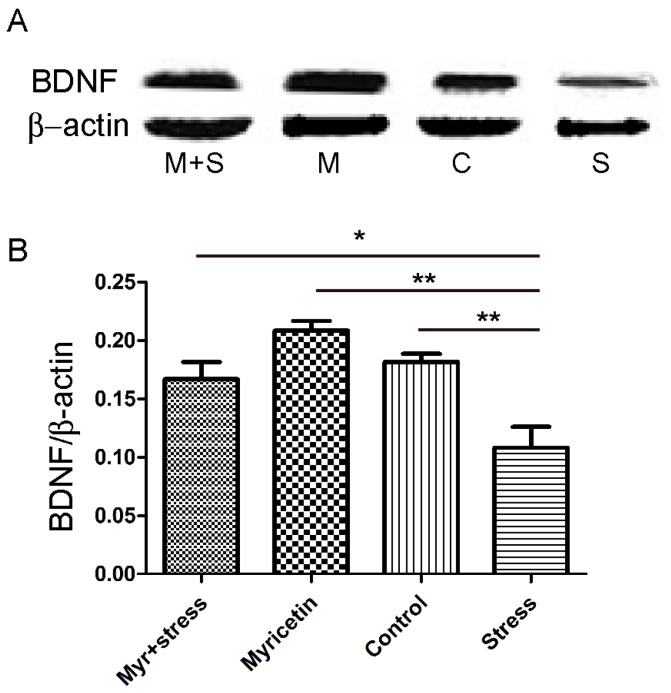
Effects of myricetin on brain-derived neurotrophic factor (BDNF) levels in the hippocampus of mice subjected to repeated restraint stress for 21 consecutive days. (**A**) The expressions of BDNF significantly decreased in the hippocampus of mice subjected to repeated restraint stress for 21 consecutive days. These effects were partially restored by myricetin administration. (M + S: Myricetin + repeated restraint stress; M: Myricetin; C: Control; S: repeated restraint stress); and (**B**) statistical analysis. Data were presented as the ratio of BDNF to β-actin. Each bar represented the mean ± SEM of three independent experiments. * *p* < 0.05, ** *p* < 0.01 respectively.

The expressions of BDNF in the hippocampus of repeated restraint stress mice were measured. As shown in Figure 4, statistical analysis revealed that repeated restraint treatment significantly reduced the BDNF levels in the hippocampus (*p* < 0.01, compared with that of control). These effects were clearly reversed by chronic administration of myricetin at 50 mg/kg (*p* < 0.05, compared with that of repeated restraint stress group). Interestingly, expressions of BDNF in myricetin alone treatment group showed a tendency of increase, however no significant differences were detected (Figure 4).

## 3. Discussion

Flavonoids are plant polyphenolic compounds which have a vast variety of biological functions, including but not limited to anti-oxidative, anti-inflammation, anti-convulsant, anti-cancer, and neuroprotection. Studies have revealed that increasing consumption of flavonoids could decrease certain human diseases [12]. Recently, a large number of natural flavonoids have been investigated as potential anti-depressants [13,14,15]. We focused on the anti-depressive potential of myricetin.

Myricetin was initially studied for its anti-apoptotic potential, but also gained recognition for its ability to protect against neurotoxicity-induced neurodegeneration and reduce cardiovascular disease [16]. More recently a crude extract from *Pilea microphylla*, in which one of the main contents is myricetin, was shown to alleviate depressant-like behavior in mice [17]. The FST and TST are well-established screening paradigms for antidepressant. The immobility displayed by rodents subjected to forced swimming, may reflect a despair state or lowered mood and are thought to mimic depressive disorders in humans. In this study, we found that myricetin at the dosage of 50 mg/kg decreased immobility time in mice subjected to repeated restraint stress, as shown in both FST and TST. In the preliminary experiment, we used the dosages of 5, 25, and 50 mg/kg to testify the effect of myricetin, only 50 mg/kg myricetin treatment exhibited an antidepressant-like behavior. Thus, we choose the dosage of 50 mg/kg myricetin in the following experiments. Further studies by the open field test confirmed that the antidepressant-like effect of myricetin is not due to any psychostimulant. Our results, thus, indicated that myricetin alleviated depressant-like behavior induced by repeated restraint stress.

The HPA axis is considered to be an important target for preventing and treatment of depression. It has been shown that chronic exposure to restraint may disrupt HPA axis and induce the elevation of plasma corticosterone. Previous studies also showed that repeated restraint stress may increase corticosterone activity and the probability of depression, which are consistent with our present findings [18,19]. So far, several studies have investigated the modulatory effects of natural components and herbal medicines on HPA axis activation [20,21,22,23]. All of the studies showed an improvement of stress-related behavioral responses, however the mechanisms of these actions are still poorly understood. It is worthy to note, we found that myricetin partially restored the increased plasma corticosterone caused by repeated restraint stress, which might at least partly contribute to the anti-depressant effect of myricetin.

In support of previous study showing that repeated restraint stress may induce robust increases in basal oxidative stress [24,25,26], we also observed that GSH-PX activities in the hippocampus decreased after repeated restraint stress. Noticeably, we found that myricetin treatment restored the GSH-PX activities to normal. It has been reported that chronic administration of corticosterone could induce oxidative damage comparable to that induced by restraint stress [27], which implicated oxidative stress as one of the major pathological mechanisms underlying chronic stress exposure and depression. Additionally, GSH-PX activity reduction probably is dependent on gluthatione levels reduction in hippocampus and increase in corticosterone levels. It is well known that oxidative stress may induce many damages in stress disorders, such as neuronal damage, impairment of neurogenesis and mitochondrial dysfunction. Thus, the anti-depressant effect of myricetin may at least partially attribute to its anti-oxidative action. In the present study, we could not find a significant decrease, although has a tendency, in SOD activity in hippocampus following chronic stress, which might be attributed to compensatory response to chronic stress.

BDNF plays a key role in the survival and growth of the neurons [28]. To date, many studies have revealed that several neurotrophic factors, including but not limited to BDNF, and related signaling transduction pathways might be involved in the pathogenesis of depression [29,30]. In support of previous study, we found that expressions of BDNF decreased in the hippocampus of mice subjected to repeated restraint stress. The hippocampus belongs to limbic structure, which plays a critical role in the control of learning and memory and in the regulation of the HPA axis. Previous studies have reported that stress may inhibit the expressions of BDNF in the limbic structures, and these effects could be reversed by chronic antidepressant treatment [31]. Our data demonstrated that chronic administration of myricetin restored hippocampal BDNF protein levels in mice subjected to repeated restraint stress. In the present study, we did not investigate BDNF levels in prefrontal cortex and amygdala. It is well known that amygdala and prefrontal cortex play an important role in mediating depressant behaviors, we cannot discard the idea that expressions of BDNF might be altered in those regions.

We did not measure the monoamines levels in the present study. However, we cannot discard the idea that myricetin-mediated antidepressant effects may be partly attributed to the alterations of monoamines levels. Monoamines are correlated with depression. Several studies have reported that chronic stress may induce depressive-like behavior by inhibition of serotonin and dopamine neurotransmission [5,32]. It is well known that some natural compounds may target on serotonergic and dopaminergic system. It has been reported that chronic administration of carvacrol may increase serotonin and dopamine levels in the hippocampus and prefrontal cortex [33]. Naringenin also revealed an antidepressant property by elevation of serotonin and norepinephrine concentration [34]. Recent studies demonstrated that quercetin, an extract of *Tagetes lucida* which has the similar structure with myricetin, may modulate the release and re-uptake of serotonin [35]. Another study also reported that myricetin may directly inhibit the activity of serotonin *N*-acetyltransferase, which catalyzes the conversion of serotonin to *N*-acetylserotonin [36]. Thus, modulation of serotonin may also play an important role in antidepressant effect of myricetin.

In summary, our findings indicate that administration of myricetin attenuated the depressant-like behaviors in mice exposed to repeated restraint stress. The underlying mechanisms might be partially mediated by restoring the BDNF levels and might be attributed to myricetin-mediated anti-oxidative stress in the hippocampus.

## 4. Materials and Methods

### 4.1. Animal Preparation

All procedures were carried out in accordance with the National Institute of Health Guide for Care and Use of Laboratory Animals. Adult male C57BL/6 mice weighting 25–30 g were obtained from Vital River Company and housed with a 12 h light/dark cycle (6:00–18:00) and free access to food and water. After habituation for at least one week, mice were randomly divided into four groups with 15 animals each to receive different treatments: chronic stress stimuli with myricetin injection (stress + myricetin), chronic stress stimuli with vehicle injection (stress + vehicle), and controls treated with myricetin (control + myricetin) or vehicle (control + vehicle). Mice receiving chronic stress stimuli were restrained daily for 4 h/day and for consecutive 21 days in well ventilated plexiglass tubes without access to food and water. Control animals were housed in their home cage without disturbing. Myricetin with a dose of 50 mg/kg was injected intraperitoneally at 60 min prior to daily restraint for consecutive 21 days. Myricetin used in this experiment was purchased from Sigma-Aldrich (Shanghai, China), which was dissolved in DMSO and diluted in 0.9% NaCl solution.

### 4.2. Forced Swimming Test (FST) and Tail Suspension Test (TST)

After treated for 21 days, individual mice in each group were forced to swim in an open cylindrical container (diameter 23 cm, height 30 cm), containing 20 cm deep water at 25 °C. The duration of immobility, defined as the absence of escape-oriented behaviors, was measured for 5 min. The same animals were subjected to tail suspension test during the next day. Before testing, the mice were transported to testing area and were allowed to adapt for 1 h. Testing animals were then suspended 70 cm above the desktop by surgical tape sticked on the tails 1 cm away from the tips. The total immobility time was recorded during 10 min test period. Immobility is defined as not having limbs and body movements, except for respiration [37].

### 4.3. The Open Field Test

The open field test was conducted to assay the animals’ general locomotor activity and anxiety. The test was carried out in a square arena (27.3 × 27.3 × 20.3 cm^3^). When doing this experiment, the mouse was initially placed in the center of the arena. After habituation to the environment for 5 min, the total distance traveled of the mice in 10 min and the percentage time spent in the center of the field were recorded.

### 4.4. Biochemical Analysis of Hippocampal Homogenate

At the end of the restraint, mice in each group were sacrificed by decapitation. The brain was removed and the hippocampus was quickly dissected on an ice-cold surface. Brain tissue was shortly homogenized in cold 0.9% NaCl solution to give 10% homogenate (*w*/*v*). After being centrifuged at 2500 rpm/min for 10 min at 4 °C to remove nuclei and debris, the supernatants were separated for further analysis.

The superoxide dismutase (SOD) and glutathione peroxidase (GSH-PX) activity assay kits were purchased from Nanjing Jiancheng Bio-engineering Institute (Nanjing, China). Total SOD activity and GSH-PX activity were assayed with the guidance of the instructions described in the commercial kits [38]. One unit of SOD activity is calculated by the amount of enzyme in each ml of the reaction solution at 50% SOD inhibition at 37 °C. One unit of GSH-PX activity is calculated by the net amount of enzyme capable of hydrolyzing 1 μmol of GSH (Glutathione) per min at 37 °C. Protein concentration was determined in hippocampal homogenates using bovine serum albumin as a standard.

### 4.5. Corticosterone Assay

Mice in each group were sacrificed by decapitation immediately after the last day of restraint and their trunk blood were collected in tubes with EDTA. The blood was centrifuged at 1000 rpm/min for 15 min at 4 °C and the plasma was stored at −70 °C until used. Plasma corticosterone levels were determined by an enzyme linked immunosorbent assay (ELISA) [39].

### 4.6. BDNF Levels in Hippocampus of Repeated Restraint Stress Mice

At the end of the restraint, mice in each group were sacrificed. The hippocampus in each mouse was isolated immediately for Western blotting test. The protein concentration was measured by a bovine serum albumin (BSA) assay. 12% SDS-PAGE was used to separate 20 μg of total protein. The protein was transferred to polyvinylidene fluoride (PVDF) membranes by electroblotting. The protein membrane was incubated in the primary antibody (rabbit anti-BDNF (1:1000) at 4 °C overnight) after blocking in 1% BSA for 2 h and then in the secondary antibody, HRP-conjugated goat anti-rabbit IgG (1:5000), for 1 h. Bound antibodies were detected by an enhanced chemiluminescence detection reagent. Band intensities were measured by the Quantity one imaging software (Bio-Rad, Hercules, CA, USA).

### 4.7. Statistics

The results were represented as mean ± SEM. Regular two-way ANOVA and Bonferroni post-test was performed using GraphPad 5.0 software (GraphPad Corp, SanDiego, CA, USA). *p* < 0.05 were considered to be significant.
